# Refining the Management of Rheumatoid Arthritis: the Benefits of Subcutaneous Tocilizumab

**DOI:** 10.1007/s40744-014-0007-2

**Published:** 2014-12-25

**Authors:** Andra F. Negoescu, Andrew J. K. Östör

**Affiliations:** Department of Rheumatology, Addenbrooke’s Hospital, Cambridge University Hospitals NHS Foundation Trust, Hills Road, Cambridge, CB2 0QQ UK

**Keywords:** Disease-modifying agents, Interleukin-6, Rheumatoid arthritis, Tocilizumab

## Abstract

Rheumatoid arthritis (RA) is a chronic systemic autoimmune condition which affects approximately 1% of the adult population worldwide and is characterized by joint inflammation, with extra-articular features being common. Interleukin 6 (IL-6) is one of the chief pro-inflammatory cytokines found in the joints and sera of patients with RA. Increased levels of IL-6 correlate with inflammation, disease activity, and radiological damage. RA treatment should focus on minimizing the signs and symptoms of disease (pain, stiffness, and swelling of the joints) and on preventing or minimizing joint damage to preserve functionality and quality of life. The benefits of early, intensive intervention are now acknowledged, with all patients with newly diagnosed, active RA being started on methotrexate (MTX) monotherapy or combination therapy. Lack of efficacy, intolerance, and/or toxicity can lead to discontinuation of this drug, and there is a need for exploring further treatment options. In the UK, patients with persistently high disease activity who have failed at least two conventional disease-modifying agents (DMARDs) including MTX may qualify for biologic therapy. Numerous trials have shown intravenous (IV) tocilizumab (TCZ), a biologic drug targeting and inhibiting IL-6, to be effective for controlling inflammation in RA, with an acceptable safety profile. Its superiority in monotherapy when compared with other biologic agents makes it the drug of choice for patients who are intolerant or have contraindications to traditional DMARDs. However, one of the drawbacks of IV TCZ is the requirement for monthly infusions, which is inherently inconvenient for the patient and associated with increased cost. Subcutaneous (SC) TCZ has now been approved following two clinical trials which showed similar efficacy and safety compared to IV TCZ, and better efficacy compared to placebo (SUMMACTA and BREVACTA trials, respectively). Respiratory infections are the most common side effects in patients receiving SC TCZ. Advantages of SC formulations include convenience and reduced cost compared with IV therapies. Overall, patients tend to have a preference for SC over IV administration of medications. Close monitoring of patients should be undertaken in all cases, paying particular attention to the full blood count, liver enzymes, and cholesterol levels.

**Electronic supplementary material:**

The online version of this article (doi:10.1007/s40744-014-0007-2) contains supplementary material, which is available to authorized users.

## Introduction

Rheumatoid arthritis (RA) is a chronic systemic autoimmune condition characterized by joint inflammation, although extra-articular features are common. RA affects approximately 1% of the adult population worldwide and is more common in women [1, 2]. The incidence of RA in the UK is 36 per 100,000 women and 14 per 100,000 men per year [1, 2] and RA may lead to significant disability, reduced quality of life, and increased mortality [1, 3–5]. Furthermore, it has a significant impact on work productivity, with approximately one-third of patients having to leave employment within 2 years of diagnosis [6].

### Joint Involvement in Rheumatoid Arthritis

Although typically a disease of the hands and feet, RA may affect any synovial joint. Inflammation of the synovium, followed by progressive degradation of cartilage and subsequent bone erosion are the hallmark of active untreated disease.

### Systemic Effects of Rheumatoid Arthritis

In addition to joint destruction, RA can result in a variety of extra-articular manifestations including anemia, localized and generalized osteoporosis [7, 8], nodulosis, eye disease, and pulmonary and cardiovascular (CV) disease [9, 10]. Being diagnosed with RA is an independent risk factor for atherosclerosis (similar in severity to type 2 diabetes mellitus) due to chronic, systemic inflammation [11–13]. CV disease has emerged as the number one cause of mortality in patients with RA [14, 15].

### Immunopathogenesis

The articular and extra-articular manifestations of RA are caused at a molecular level by increased levels of pro-inflammatory cells, cytokines, and autoantibody production [8]. Pro-inflammatory cytokines, such as tumor necrosis factor alpha (TNF-α), interleukin 1 (IL-1), interleukin 6 (IL-6), interleukin 17 (IL-17), interferon gamma (IFN-γ), and transforming growth factor beta (TGF-β), can stimulate the release of further cytokines [16–19], leading to excessive cellular activation and migration into the synovium and sustained inflammation [16, 17, 19].

## The Role of IL-6 in Rheumatoid Arthritis

IL-6 is one of the chief pro-inflammatory cytokines found in the joints and sera of patients with RA [20–23]. A number of cell types produce and release IL-6, including activated macrophages, synovial fibroblasts, and T and B cells [8, 24–26]. Increased levels of IL-6 correlate with inflammation, disease activity, and radiological damage [8, 24, 27–30]. Furthermore, the decrease in serum IL-6 levels in the first 12 months of therapy with disease-modifying agents (DMARDs) is a powerful prognostic marker for clinical outcomes [28].

## Current Treatments for Rheumatoid Arthritis

### Non-steroidal Anti-inflammatory Drugs and Traditional DMARDs

Treatment for RA should focus on minimizing the signs and symptoms of the disease (pain, stiffness, and swelling of the joints) and on preventing or minimizing joint damage to preserve functionality and quality of life. In addition, reducing the extra-articular manifestations and implicitly reducing the premature mortality associated with the condition is critical [31, 32].

Suppression of inflammation is the central element of RA management, with remission (defined as the complete suppression of inflammation and prevention of joint destruction) being the ultimate goal of therapy [16, 19, 33]. Non-steroidal anti-inflammatory drugs (NSAIDs) may provide fast and effective relief of symptoms, but do not alter the disease course. The cornerstone of therapy, therefore, is DMARDs, which should be instituted as early as possible to prevent long-term joint damage [34–36]. For safety and efficacy reasons, patients should be frequently monitored and treatment altered accordingly, adopting a treat to target strategy [37].

Although highly effective in many cases, traditional DMARDs [such as methotrexate (MTX), sulfasalazine (SSZ), leflunomide (LEF), and hydroxychloroquine (HCQ)] can be associated with toxicity and/or intolerability, with a high impact on adherence and thus on disease control [38–41].

The benefits of early, intensive intervention are now acknowledged, with all patients with newly diagnosed, active RA being started on MTX monotherapy or combination therapy (usually with HCQ). The pre-eminent DMARD is MTX; however, lack of efficacy, intolerance, and/or toxicity can lead to discontinuation [40], and with it the need of exploring further treatment options.

### Biologic Agents

Biologic agents target specific pro-inflammatory cytokines, cells, or molecules involved in the pathogenesis of RA [16]. Five anti-TNF-α therapies (infliximab, adalimumab, etanercept, certolizumab, and golimumab) are currently licensed in Europe and the USA [16, 19]. Although TNF-inhibitors have revolutionized RA treatment over the last decade or so, there remains an area of unmet need, with up to 40% of patients failing to respond to this particular treatment [16, 19, 42]. Other disadvantages of TNF-α inhibitors include contraindications such as heart failure, chronic or recurrent infections, and demyelinating conditions, as well as side effects that can lead to treatment discontinuation (serious infections, injection site reactions (ISR), melanoma, non-melanoma skin malignancies [43], and lupus-like illness). Reactivation of latent tuberculosis (TB) infection may occur; however, this has been minimized by screening programs and the use of prophylactic anti-TB agents in those at risk.

Due to the area of unmet needs, other biologic agents have been trialed in RA. Anakinra, an IL-1 inhibitor, was one of the first biologics studied in RA [44]; however, it is no longer used due to its lower efficacy compared with other agents and the poor tolerability of daily subcutaneous (SC) injections. Rituximab (RTX), an anti-CD20 found on B cells, is now well established for the treatment of RA [45]. Further biologic agents include abatacept which inhibits the co-stimulation of T cells [46], and tocilizumab (TCZ), an IL-6 receptor antagonist [47]. Whereas RTX is administered by intravenous (IV) infusion no less frequently than 6 monthly, the latter two agents were initially administered as IV infusions; however, SC formulations are now available [48–51]. This article will specifically focus on SC TCZ including its safety, efficacy, and how it may fit in RA treatment regimens. Furthermore, this article is based on previously conducted studies and does not involve any new studies of human or animal subjects performed by any of the authors.

## Efficacy of Subcutaneous Tocilizumab in Rheumatoid Arthritis

Numerous trials have shown IV TCZ to be an effective drug for controlling inflammation in RA with an acceptable safety profile [47, 52–56]. Its superiority in monotherapy when compared with other biologic agents makes it the drug of choice for patients who are intolerant or have contraindications to traditional DMARDs [36]. However, one of the drawbacks of IV TCZ is the requirement for monthly infusions with the inherent inconvenience for the patient and cost. Two phase III clinical trials have led to the approval of SC TCZ, having shown the efficacy and tolerability of SC TCZ in RA [50, 51].

The SUMMACTA trial (ClinicalTrials.gov #NCT01194414) compared SC and IV TCZ and met its primary end point, showing that TCZ SC 162 mg weekly is non-inferior to TCZ IV 8 mg/kg in terms of efficacy [50]. The American College of Rheumatology 20 (ACR 20) response was achieved in 69.4% of patients at week 24 in the TCZ SC group, compared with 73.4% in the TCZ IV group. The difference of −4.0% (95% confidence interval [CI] −9.2 to 1.2) met the requirement for the non-inferiority of TCZ SC to TCZ IV. The ACR 50 and ACR 70 response rates at 24 weeks were also similar between groups (weighted differences of ACR 50 and ACR 70 responders at week 24: −1.8 and −3.8%, respectively).

**Fig. 1:**
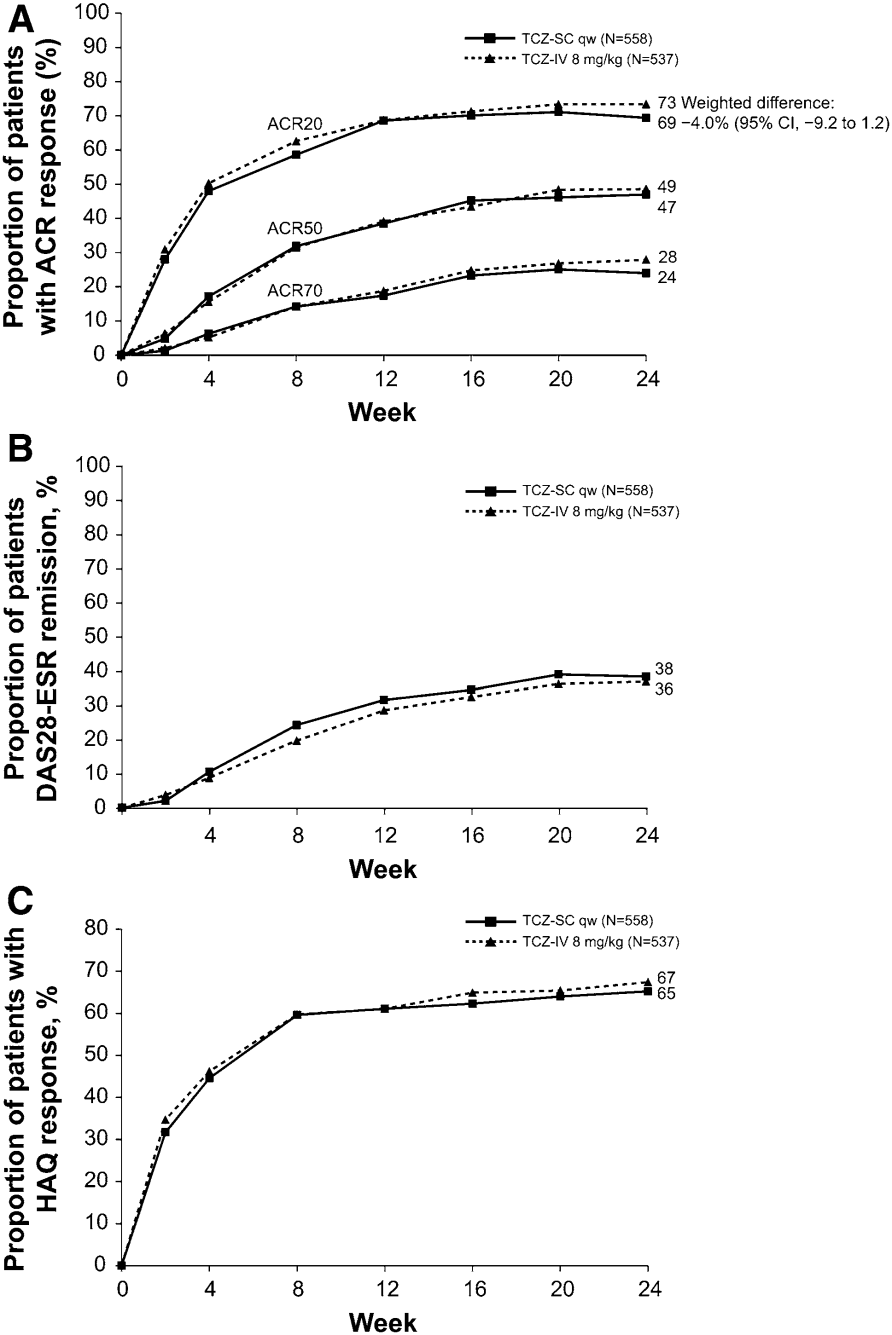
Disease activity and physical function over 24 weeks for patients in the per-protocol (PP) population. **a** Proportion of patients in the PP population treated with either subcutaneous tocilizumab (TCZ SC; *n* = 558) or intravenous tocilizumab (TCZ IV; *n* = 537) achieving 20, 50, and 70% improvements per American College of Rheumatology criteria (ACR20, ACR50, and ACR70, respectively) over 24 weeks. **b** Proportion of patients achieving remission based on disease activity score using 28 joints (DAS 28) based on erythrocyte sedimentation rate (ESR <2.6) over 24 weeks. **c** Proportion of patients achieving a health assessment questionnaire (HAQ) response (improvement of ≥0.3 from baseline) over 24 weeks. Reproduced from: Burmester et al. [50] with permission from BMJ Publishing Group Ltd. *qw* weekly

Disease Activity Score in 28 Joints (DAS 28) remission was another clinical outcome measured in the SUMMACTA trial [50]. The proportion of patients who achieved DAS 28 remission at week 24 was similar in both SC and IV groups, with a weighted difference of 0.9%. The non-inferiority of SC TCZ to IV TCZ was also demonstrated for functionality outcomes, with a weighted difference of −2.3% in the proportion of patients achieving a decrease of 0.3 or greater in Health Assessment Questionnaire Disability Index (HAQ-DI) from baseline (Fig. 1).

The BREVACTA trial (ClinicalTrials.gov #NCT01232569) studied the efficacy and safety of SC TCZ compared to SC placebo in patients with RA who had an inadequate response to traditional DMARDs [51]. The study met its primary end point, showing the superiority of SC TCZ (162 mg every 2 weeks) to SC placebo in achieving ACR 20 response at week 24 [60.9 and 31.5%, respectively; weighted difference 29.5% [95% CI 22.0–37.0; *P* < 0.0001]). In a similar trend, ACR 50 and ACR 70 response rates at week 24 were significantly higher in the SC TCZ group (27.9% [95% CI 21.5–34.4; *P* < 0.0001] and 14.8% [95% CI 9.8–19.9; *P* < 0.0001], respectively). DAS 28 remission (defined as DAS 28 < 2.6) was significantly higher at week 24 in the SC TCZ group compared to the placebo group (weighted difference 28.6% [95% CI 22.5–35.2; *P* < 0.0001]). The mean change from baseline at week 24 in the total Sharpe score was lower in the SC TCZ group compared to the placebo group (0.62 ± 2.692 vs. 1.23 ± 2.816), with a significant difference in the erosion score and a non-statistically significant difference in the joint space narrowing score.

## Safety of Subcutaneous Tocilizumab

The safety of SC TCZ was also assessed in the SUMMACTA (Table 1) and BREVACTA studies. In the SUMMACTA trial a similar safety profile was found between the IV and SC groups, with the exception of a higher incidence of ISR for the SC group, as would be expected [168 ISR in the SC group (10.1% of patients) vs. 94 ISR in the IV group (2.4%)]. However, ISR were all deemed non-serious and did not require treatment interruptions or discontinuation [50]. The most common side effect described in the studies was infection, particularly upper respiratory tract infection (7.3% TCZ SC and 11.6% TCZ IV). Serious infections were rare, but reported in both groups (pneumonia—two cases in each group). Septic arthritis occurred in two patients in the IV group, with one case progressing to sepsis and death. No deaths were reported in the SC group (0/631; 1/631 in the IV group). The most common cause for study discontinuation due to side effects was infection in both groups (1.1% TCZ SC and 1.3% TCZ IV).

**Table 1:** Safety summary (safety population)

	Tocilizumab SC 162 mg qw (*n* = 631) 289.82 PY	Tocilizumab IV 8 mg/kg q4w (*n* = 631) 288.39 PY
AE		
Total AE, *n*	1,747	1,697
Patients with >1 AE, *n* (%)	481 (76.2)	486 (77.0)
Discontinuation due to AE, *n* (%)	30 (4.8)	42 (6.7)
SAE		
Total SAE, *n*	34	43
Patients with >1 SAE, *n* (%)	29 (4.6)	33 (5.2)
SAE per 100 PY (95% CI)	11.73 (8.12–16.39)	14.91 (10.79–20.08)
SI		
Total SI	9	9
Patients with >1 SI, *n* (%)	9 (1.4)	9 (1.4)
SI per 100 PY (95% CI)	3.11 (1.25–5.89)	3.47 (1.66–6.38)
Serious hypersensitivity reactions^a^, *n* (%)	2 (<1)	3^b^ (<1)
ISR		
Patients with ISR, *n* (%)	64 (10.1)	15 (2.4)
ISR, *n*	168	94
Erythema, *n* (%)	28 (4.4)	5 (0.8)
Pain, *n* (%)	12 (1.9)	5 (0.8)
Pruritus, *n* (%)	14 (2.2)	0(0)
Hematoma, *n* (%)	5 (0.8)	5 (0.8)
Dose interruption or study withdrawal because of ISR, *n*	0	0
Death, *n* (%)	0 (0)	1 (<1)

Reproduced from Burmester et al. [50] with permission from BMJ Publishing Group Ltd.

*AE* adverse event, *ISR*_*r*_ injection site reaction, *IV* intravenous, *PY* patient-years, *qw*_*r*_ every week, *q4w*_*r*_ every 4 weeks, *SAE* serious adverse event, *SC* subcutaneous, *SI* serious infection

^a^Serious hypersensitivity was defined as an SAE occurring during or within 24 h of the injection or infusion, excluding ISR, and evaluated as ‘related’ to study treatment by the investigator

^b^Of the three events in the tocilizumab IV group, one was cellulitis and one was retinal artery occlusion; these two events were not considered consistent with a serious hypersensitivity reaction

The use of TCZ leads to a strong decrease in C-reactive protein: this makes the clinical evaluation of patients with a possible septic disorder much more difficult. Other well-recognized side effects of TCZ are its effects on liver function tests (LFTs), neutrophils, and cholesterol levels. Raised LFTs were seen in the SUMMACTA, as well as BREVACTA trials. No differences between groups were observed for alanine-transaminase (ALT) and aspartate-transaminase (AST) rises from normal to a value more than three times the upper limit of normal which occurred in 4.8 vs. 5.1% of patients for ALT and 1 vs. 1.3% for AST, in the SC and IV TCZ groups, respectively [50].

Neutropenia >1,000 × 10^9^/L was reported in a slightly higher proportion in the SC group compared to the IV group (32.8 vs. 23.3%); however, there was no difference between groups with regard to severe neutropenia (<1,000 × 10^9^/L), 2.7 and 3.2%, respectively. Increases in total cholesterol levels were also more frequent in the SC group compared to the IV group (23.8 vs. 20.6%), but with similar proportions between groups with regard to rises in low-density lipoprotein cholesterol, high-density lipoprotein cholesterol, and triglyceride levels [50].

The BREVACTA trial showed similar safety findings, with respiratory infections being the most common side effects, 6.4% in each group [51]. The most common ISR were erythema, pruritus, and pain, and the proportion was higher in the SC TCZ group compared to the placebo group (7.1 and 4.1%, respectively). No anaphylaxis or serious hypersensitivity reactions were reported. Only 6.3% of patients discontinued SC TCZ due to adverse events (most commonly due to infections and raised LFTs).

Infection was reported as the most common serious adverse event (2.1% in the SC TCZ group vs. 1.8% in the placebo group). Three deaths (3/437) were reported by week 24, all in the SC TCZ group (one death attributable to hemophilus influenza sepsis, one to sepsis with pancytopenia likely of gastrointestinal origin, and one to lower respiratory tract infection). No gastrointestinal perforation was reported up to week 24, although one event of diverticular hemorrhage was described in the SC TCZ group, in a patient with a history of diverticular bleeding. All patients were screened for TB and those with active TB were excluded.

In patients who experienced elevated LFTs, most had an increase less than three times the upper limit of normal, with shifts occurring more frequently in the SC TCZ group compared to the placebo group (33 vs. 13%). Patients experienced a decreased neutrophil count (>1,000/mm^3^) in a higher proportion in the SC TCZ group (16.7 vs. 3.7%), while severe neutropenia (<1,000/mm^3^) was only registered in the SC TCZ group (3.7%). No events of severe thrombocytopenia (<50,000/mm^3^) occurred. The proportion of patients with cholesterol level shifts from <200 mg/dL at baseline to ≥200 mg/dL was higher in the SC TCZ group compared to the placebo group (45 and 14%, respectively). Increases in low-density lipoprotein cholesterol and triglyceride levels also occurred more frequently in the SC TCZ group compared to the placebo group.

## The Current Rheumatoid Arthritis Treatment Pathway and Subcutaneous Tocilizumab

Current guidelines recommend that newly diagnosed patients with moderate to severe RA are commenced on a combination of DMARDs, usually hydroxychloroquine and methotrexate or possibly MTX monotherapy [34, 36]. The most important element of treatment, however, is that patients are seen and started on immunosuppression as early as possible, to optimize outcomes [57].

**Fig. 2:**
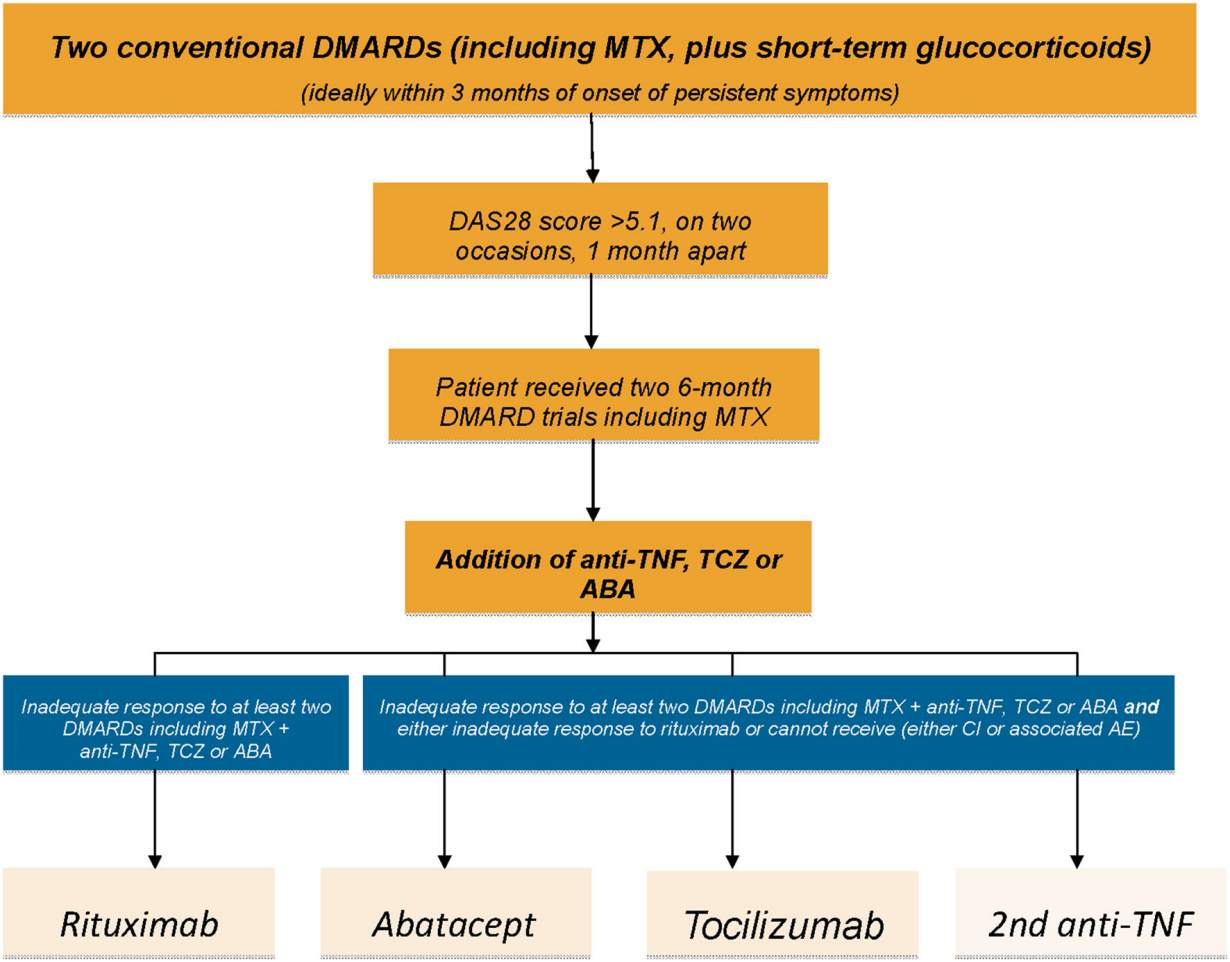
NICE rheumatoid arthritis clinical treatment pathway. *ABA* abatacept, *AE* adverse events, *CI* contraindications, *DAS* 28 Disease Activity Score in 28 Joints, *DMARD* disease-modifying antirheumatic drug, *MTX* methotrexate, *NICE* National Institute for Health and Care Excellence, *RA* rheumatoid arthritis, *TCZ* tocilizumab, *TNF* tumor necrosis factor

In the UK, patients with persistently high disease activity (two DAS 28 scores >5.1 at least 1 month apart) who have failed at least two conventional DMARDs trialed (including MTX) for a minimum of 6 months at the standard dose (or less if treatment resulted in side effects) may qualify for biologic therapy (see Fig. 2). According to the National Institute for Health and Care Excellence (NICE) and European League Against Rheumatism (EULAR) recommendations, three classes of biologic agents can be given as first line to patients with active RA:

1. Anti-TNF α, with a choice of five agents (adalimumab, etanercept, golimumab, certolizumab, and infliximab), all of which are available by SC apart from infliximab which is administered by IV infusion;
2. Abatacept (available both by IV and SC);
3. TCZ, (available both by IV and SC).

NICE guidelines follow European Commission and Food and Drug Administration (FDA) approval of SC TCZ for the treatment of moderate to severe RA in patients who are either intolerant to or have failed to respond to other RA treatments, in April and October 2013, respectively. This approval made TCZ the first anti-IL-6 receptor biologic available as SC and IV formulations for both mono and combination therapy with MTX [58]. TCZ can also be given as third line for patients with RA, after failure/intolerance to anti-TNF α and RTX, or as second line, if contraindications to RTX exist (see Fig. 2).

## Practical Aspects of Subcutaneous Formulation

The advantages of SC formulations include convenience and reduced cost compared with IV therapies. Time off work or usual activities to attend pre-booked IV infusions requires significant infrastructure and is not patient focused. Self-administration (following appropriate training) is ideal for many patients such as those with full-time work commitments, child care issues, and who travel frequently. Overall, patients tend to have a preference for SC over IV administration of medications [59–61]. However, there are patients for whom IV administration is more suitable. These include patients with extensive hand deformities or who have needle phobia, and those where there is concern regarding drug compliance.

Regardless, close monitoring of patients should be undertaken in all cases paying particular attention to the full blood count, liver enzymes, and cholesterol levels. The British Society for Rheumatology (BSR) has published national guidelines for patients receiving IV TCZ, although local policies may differ [62]. The IV TCZ guidelines for patients with RA will be used as a proxy for monitoring the SC TCZ formulation. In addition, as neutropenia and cholesterol level shifts are more frequent in the SC group, we suggest that closer monitoring of these parameters should be performed for patients prescribed SC formulation. The summary of recommendations is as follows:

1. Baseline fasting lipid profile (if abnormal, treatment should be given in accordance with local guidelines); lipid profile should be repeated in 3 months and treatment instituted/altered if appropriate.
2. Four-weekly monitoring of the absolute neutrophil count for the first 6 months, then less frequently if severe neutropenia does not occur.
3. Four-weekly monitoring of LFTs for the first 6 months (and every 2–3 months thereafter if stable), caution when using other hepatotoxic drugs, and education with regard to reducing alcohol consumption.
4. Four-week interruption of TCZ prior to elective joint replacement surgery, to reduce the risk of post-operative infection. TCZ can be restarted once infection is excluded and the wound has healed.
5. TCZ should be stopped at least 3 months prior to planned conception and should not be given to women who breastfeed.
6. Annual influenza vaccine and pneumococcal vaccination are recommended and should be encouraged, while live attenuated vaccines are contraindicated.
7. TCZ should be used with caution in patients who have a history of diverticulitis, or are taking corticosteroids and/or NSAIDs, due to the risk of gastro-intestinal perforation.

## Tocilizumab Monotherapy in Rheumatoid Arthritis

It would appear that there is an advantage in using TCZ as monotherapy compared with other biologic agents [63]. This has particular relevance as MTX is poorly tolerated in a substantial number of patients [40].

The AMBITION trial (ClinicalTrials.gov #NCT00109408) compared TCZ monotherapy (8 mg/kg every 4 weeks) with MTX monotherapy in patients with active RA (including a high proportion of patients with early disease) over 24 weeks [52]. Results showed superiority of TCZ monotherapy, with a significant improvement in disease signs and symptoms. The weighted difference for ACR 20 response at week 24 was 0.19 (95% CI 0.11–0.27, *P* < 0.001). TCZ was also superior to placebo at week 8 (ACR 20 remission: 55.6 vs. 13.1%) with a weighted difference of 0.43. The superiority of TCZ was also seen in MTX-naive patients (ACR 20 remission: 53.7 vs. 68.6%; ACR 50 remission: 33.2 vs. 45%; ACR 70 remission: 14.2 vs. 27.2% in the MTX and TCZ groups, respectively).

DAS 28 at week 24 also improved in a higher proportion in the TCZ group compared to MTX group (adjusted mean change from baseline: −3.31 vs. −2.05) and the proportion of patients in remission at week 24 (DAS 28 < 2.6) was higher in the TCZ group (32 vs. 4%). An increase in hemoglobin (Hb) levels was seen in the TCZ group (by 1.19 g/dL from baseline), more so than in the MTX group, with an increase only by 0.10 g/dL at week 24. Normalization of mean Hb (from a level less than the lower limit of normal at baseline) occurred in the TCZ group by week 6, and the effect was maintained to week 24. This effect was not seen in the MTX group. Patients in the TCZ group also had a greater improvement in physical function as measured by HAQ-DI (−0.7 vs. −0.5 from baseline). This is the only study to date which has shown clinical superiority of a biologic agent given as monotherapy compared with MTX monotherapy [52].

In addition, the recently published ADACTA trial, a head-to-head monotherapy trial comparing TCZ with adalimumab (ADA) in patients with active RA who had failed or developed side effects to MTX, showed superiority of TCZ monotherapy (DAS 28 mean change from baseline: −3.3 in the TCZ group vs. −1.8 in the ADA group, difference −1.5, 95% CI −1.8 to −1.1; *P* < 0.0001) [64]. Starting with week 16, more patients in the TCZ group received ACR and EULAR remission [33], compared to the ADA group (in ADACTA). Physical function as assessed by the HAQ-DI score improved more significantly in the TCZ group than in the ADA group (change from baseline to week 24: −0.7 vs. −0.5; difference between adjusted means: −0.2, 95% CI −0.3 to 0.0; *P* = 0.0653). The proportion of patients with HAQ-DI improvement of at least 0.22 from baseline to week 24 was higher in the TCZ group compared to the ADA group (56.4 vs. 51.2%). This data suggest that TCZ is a preferable biologic agent for patients with active RA who are intolerant to traditional DMARDs [36].

The MUSASHI trial (ClinicalTrials.jp #JapicCTI-101117), a recent phase III study on Japanese patients, assessed the efficacy and safety of SC vs. IV TCZ monotherapy in patients with RA [65]. The study met its primary end point, demonstrating the non-inferiority of TCZ SC monotherapy to TCZ IV monotherapy. The ACR 20 response rate at week 24 was achieved in 79.2% (95% CI 72.9, 85.5) of patients in the SC group and 88.5% (95% CI 83.4, 93.5) in the IV group (weighted difference −9.4% [95% CI −17.6, −1.2]).

ACR 50 and ACR 70 response rates at week 24 were also similar between groups. DAS 28-erythrocyte sedimentation rate (ESR), Clinical Disease Activity Index (CDAI), and Boolean Index remission rates at week 24 were 49.7, 16.4, and 15.7%, respectively, in the SC group, and 62.2, 23.1, and 16.0%, respectively, in the IV group. DAS 28-ESR low disease activity at week 24 was achieved in a higher proportion in the IV group (82.1% [95% CI 76.0, 88.1]) than in the SC group (65.4% [95% CI 58.0, 72.8]). Physical function improvement was assessed by HAQ-DI and defined as a change of −0.3 units from baseline at week 24. This was 56.6% (95% CI 48.9, 64.3) and 67.9% (95% CI 60.6, 75.3) in the SC and IV groups, respectively.

The safety profiles were comparable between groups, with the exception of ISRs, which occurred more frequently in the SC group than in the IV group. Over 24 weeks, AEs occurred in 89.0% (154/173) and 90.8% (157/173), SAEs in 7.5% (13/173) and 5.8% (10/173), adverse drug reactions in 83.2% (144/173) and 86.1% (149/173) of patients, and serious adverse drug reactions in 3.5% (6/173) and 5.8% (10/173) of patients in the SC and IV groups, respectively. No deaths or malignancies were reported.

Infections were reported in 41.6 and 45.1% of patients in the SC and IV groups, respectively. Nasopharyngitis was the most common event (17.9% in the SC group and 20.8% in the IV group). Serious infections (herpes zoster, pneumonia, cellulitis, gastroenteritis) occurred in 1.2% of patients in the SC group and in 2.9% of patients in the IV group. ISRs were reported in 12.1% of patients in the SC group and in 5.2% in the IV group (placebo injection). All ISRs were mild and no cases resulted in discontinuation from the study. One patient (0.6%) in the IV group had an anaphylactic reaction after the second infusion and was withdrawn from the study. There were no cases of serious hypersensitivity in the SC group. The proportion of patients experiencing elevations in lipid levels and LFTs was similar between groups. Increases in total cholesterol from <200 mg/dL at baseline to ≥200 mg/dL occurred in 56.1 and 53.7% of cases in the SC and IV groups, respectively. Grade 1 and 2 shifts in ALT and AST were reported in 22.5% of patients in both SC and IV groups (ALT) and 12.8 versus 18% in the SC and IV groups, respectively (AST). Grade 3 shifts were rare (1 patient in the SC group and 2 patients in the IV group for ALT, 1 patient in the SC group for AST). There were no grade 4 shifts in LFTs reported. A proportion of 2.9% of patients experienced grade 3 neutropenia (500–1,000 cells/mm^3^) in each group, with 1 patient in the SC group being withdrawn from the study. No grade 4 neutropenia (<500/mm^3^) was reported.

## Conclusion

Following the success of IV TCZ, the arrival of SC TCZ is a welcome addition to the arsenal to combat the morbidity and premature mortality associated with RA. The efficacy of SC TCZ in both monotherapy and combination therapy and the acceptable safety profile are reassuring; however, longer-term data are required. Certain groups of patients may benefit, especially those intolerant to traditional DMARDs. Increasingly, SC TCZ will be incorporated into management paradigms to optimize the outcomes for all RA patients.

## Electronic supplementary material

Below is the link to the electronic supplementary material.

Supplementary material 1 (PDF 186 kb)
