# Supplementary material for: Refining the Management of Rheumatoid Arthritis: the Benefits of Subcutaneous Tocilizumab
Source: Rheumatol Ther. 2014 Dec 25;2(1):17–31. doi: 10.1007/s40744-014-0007-2 (PMC4883250; doi:10.1007/s40744-014-0007-2)
Supplement: Supplementary file 1 — Supplementary material 1 (PDF 186 kb) [file 40744_2014_7_MOESM1_ESM.pdf]

Rheumatology and Therapy
OPEN ACCESS
PEER REVIEWED SUMMARY SLIDE

- Treatment in rheumatoid arthritis (RA) should focus on minimizing the signs and symptoms of disease (pain, stiffness, and swelling of the joints) and on preventing joint damage.
- In the UK, patients with persistently high disease activity who have failed at least two conventional disease-modifying agents (DMARDs) including methotrexate may qualify for biologic therapy.
- Tocilizumab (TCZ) is a biologic drug which inhibits interleukin 6, an interleukin involved in the RA inflammatory pathway.
- Its superiority in monotherapy when compared to other biologic agents makes it the drug of choice for patients who are intolerant or have contraindications to traditional DMARDs.
- Subcutaneous formulation of TCZ has now been approved and it has a similar safety and efficacy profile to intravenous TCZ, with the advantages of being more convenient for patients and less costly.

This summary slide represents the opinions of the authors. No funding or sponsorship was received for this study or publication of this article. For a full list of acknowledgments and conflicts of interest for all authors of this article, please see the full text online. Copyright © The Author(s) 2014. Creative Commons Attribution Noncommercial License (CC BY-NC).
